# Cadmium Accumulation and Depuration in the Muscle of Prussian Carp (*Carassius gibelio* Bloch) after Sub-Chronic Cadmium Exposure: Ameliorating Effect of Melatonin

**DOI:** 10.3390/ani11082454

**Published:** 2021-08-20

**Authors:** Ewa Drąg-Kozak, Ewa Łuszczek-Trojnar, Magdalena Socha

**Affiliations:** 1 Department of Animal Nutrition and Biotechnology, and Fisheries, University of Agriculture in Krakow, Al. Adama Mickiewicza 24/28, 30-059 Kraków, Poland; ewa.trojnar@urk.edu.pl; 2 Department of Animal Physiology and Endocrinology, University of Agriculture in Krakow, Al. Adama Mickiewicza 24/28, 30-059 Kraków, Poland; magdalena.socha@urk.edu.pl

**Keywords:** fish, melatonin, muscle, accumulation, heavy metals, depuration

## Abstract

**Simple Summary:**

Rapid urbanization and industrialization has resulted in substantial contamination of various ecosystems, especially aquatic environments with heavy metals. Heavy metals are classified as either essential (iron, zinc, or copper) or non-essential (cadmium, lead, or mercury) for organisms. Cadmium is a toxic, cancerogenic, and mutagenic metal, occurring as anthropogenic contamination in aquatic environments. The level of cadmium uptake in animals depends on the rate at which they are accumulated and eliminated. Exceeding the permissible levels of cadmium in fish muscle may pose risks for human health in the case of contaminated fish consumption. The aim of the present study was to evaluate the influence of melatonin on cadmium accumulation and elimination in fish muscle. Prussian carps were exposed to two doses of cadmium in the presence or without the melatonin implants. This is the first study to report that melatonin co-administration can effectively protect fish from the accumulation of cadmium in muscle tissue, improve the accumulated cadmium depuration from muscle, and prevent disturbance of the concentration of essential metals in fish body.

**Abstract:**

The aim of this study was to investigate the bioaccumulation of cadmium in the muscle tissue of Prussian carp during 7 and 13 weeks of exposure to different concentrations of this metal in water (0.4 and 4.0 mg/L), and the depuration of cadmium from muscle during the following 6-week depuration period in the presence of melatonin implants. Furthermore, the relationship between cadmium accumulation and the levels of essential bioelements (copper, zinc, iron) in muscle was evaluated, as well as the bioconcentration factor of cadmium. Heavy metal concentration was determined using atomic absorption spectrometry. Cadmium accumulation in fish muscle increased with the duration of exposure. Cd concentrations exceeded the permissible levels for human consumption in groups exposed to the higher concentration of this metal. Moreover, a significant increase of Zn and Fe levels in the muscle was observed. In the fish that received melatonin implants and were exposed to Cd, its level in the muscle was significantly lower. The depuration of accumulated cadmium depended mainly on the duration of the elimination period. This is the first study to report that melatonin co-administration can effectively protect the fish from the accumulation of cadmium in muscle tissue and changes in trace metal levels.

## 1. Introduction

Environmental pollution is one of the main challenges of today’s world. Heavy metal contamination poses a threat to the environment and raises serious concerns. Rapid urbanization and industrialization have resulted in substantial contamination of various ecosystems with heavy metals [1].

Heavy metals are classified as essential or non-essential in terms of their role in biological systems [2]. Some metals have important physiological and biochemical functions in the body as they can be components of biomolecules, such as enzymes, which catalyze biochemical reactions. A deficiency or excess of those metals may disrupt metabolism, which in turn can lead to various diseases. Essential heavy metals include zinc (Zn), copper (Cu), and iron (Fe). There are also heavy metals that are toxic to living organisms. They include, for instance, cadmium (Cd), lead (Pb), and mercury (Hg) [3]. Heavy metals such as nickel (Ni), chromium (Cr), Pb, Cd, and Hg are regarded (classified) as priority hazardous substances (pollutants) in many countries [4]. Surface waters are particularly vulnerable to pollution as they receive effluents and rainwater runoff [1]. The contamination of water resources with heavy metals is a critical environmental issue due to the negative impact of those metals on plants, animals, and human health. The accumulation of heavy metals in freshwater and sea fish depends on numerous factors, both environmental (water chemistry, salinity, temperature, pH, hardness) and biological (species, size, age, sex, stage of sexual maturity and diet) [5]. Practically all heavy metals can be potentially toxic to fauna and flora, depending on their dose and duration of exposure. They are persistent in the environment, accumulate in living organisms, and are transferred from lower to higher trophic levels in the food chain, thus undergoing biomagnification. The level of uptake of heavy metals in fauna and flora depends on the rate at which they are accumulated and eliminated. As shown by Djedjibegovic et al. [6], fish are an important and recommended part of a healthy human diet. Thus, contamination of sea and freshwater fish with metals is a serious environmental issue. The World Health Organization (WHO) and the Food and Agriculture Organization of the United Nations (FAO) state that monitoring eight metals in fish, i.e., mercury, cadmium, lead, arsenic (As), copper, zinc, iron, and tin (Sn), is obligatory [7]. Environmental quality standards for cadmium in inland surface water range from <0.08 to 0.25 mg/L depending on water hardness classes [4]. Even low Cd concentrations may involve toxic effects in living organisms. The bioaccumulation of Cd in food chains is a source of concern as it may have an adverse impact on human and animal health. Cadmium accumulates mainly in the kidney. It impairs renal function and can cause kidney cancer. It has also been found that the accumulation of Cd in bone tissue leads to the very painful Itai-Itai disease [8]. Cadmium can also cross the blood–brain barrier and cause Alzheimer’s and Parkinson’s diseases [9].

In fish, the bioconcentration and biomagnification of Cd may disrupt numerous physiological processes, even at low exposure concentrations, as the metal integrates into essential protein synthesis reactions [10]. Therefore, it is very important to better understand the bioaccumulation potential of cadmium in living organisms.

The environmental impact of heavy metals on living organisms can be demonstrated by using the bioconcentration factor (BCF), which is used to assess the bioaccumulation potential of chemicals [10]. Information on the BCF is required, for instance, under the Registration, Evaluation, Authorization, and Restriction of Chemicals (REACH) Regulation, which regulates the use of chemicals in the European Union [10].

Fish are excellent bioindicators of the relative health of aquatic ecosystems due to their capacity to accumulate persistent pollutants [11]. Aquatic organisms can accumulate chemicals in two ways: directly from the environment (through the skin or respiratory surface—gills) or indirectly (by collecting and concentrating a chemical from food). Numerous studies have shown that an increase in the concentration of certain metals in the body affects the accumulation of other metals. Interactions between metals are associated with their competitive uptake from the environment and their different distribution in tissues. The interactions can be additive, synergistic, or antagonistic. Therefore, the impact of various mixtures of metals in the environment on the survival of fish may vary [12]. The toxic effects of cadmium manifest in organisms when the metal substitutes other metal ions in essential micro- and macro-elements (mainly Ca^2+^, Zn^2+^, Cu^2+^, and Fe^2+^) that are part of metalloenzymes, and as a result of its very strong affinity to biological structures containing an –SH group, including proteins, enzymes, and nucleic acids [13]. Over the last few years, increasing attention has been given to the interactions between toxic metals and essential bioelements in the body. It has been shown, among other things, that cadmium poisoning disrupts the homeostasis of biometals, leading mainly to their secondary deficiency [14].

Once cadmium enters the body, it can cause various cytotoxic reactions. It may disrupt metabolic pathways in cells or cause the production of reactive oxygen species (ROS), which affect various processes in cells and may impair the functioning of the membrane system [15]. A study by Al-Sawafi et al. [16] found histological changes in the skeletal muscle of zebrafish (*Danio rerio*) following exposure to cadmium, which were manifested by swelling, coagulation necrosis accompanied by hemocyte infiltration, as well as high necrosis.

It has been reported that some antioxidants present in the body may prevent various types of tissue damage caused by Cd [17]. Melatonin (Mel) has strong antioxidant potential and is able to maintain tissue redox homeostasis [18,19]. Mel is an indolamine which has been identified in all body fluids and in several extra-pineal sites, including the skin, gastrointestinal tract, liver, kidneys, immune system, and skeletal muscles [20]. The potential of Mel to prevent muscle damage caused by various chemical agents, such as heavy metals, has hardly been investigated and the few studies that explored this issue were carried out on mammals [18]. The impact of Mel on the accumulation of cadmium in fish muscle is completely unknown. Therefore, the aim of the present study was to determine whether melatonin, which is a known antioxidant and free-radical scavenger, can prevent the accumulation of cadmium in the muscle of female Prussian carp exposed to the metal. An additional objective of the study was to examine the impact of cadmium and/or melatonin on the accumulation (concentration) of essential bioelements such as copper, zinc, and iron in the muscle of the fish.

## 2. Materials and Methods

### 2.1. Animals

The experimental treatments were conducted on three 343 Prussian carp (*Carassius gibelio* B.) females aged 3 years (mean body weight—204.65 ± 12.58 g, body length—23.25 ± 0.49 cm) from the Experimental Station of the Department of Animal Nutrition and Biotechnology, and Fisheries of the University of Agriculture in Krakow, Poland. The fish were acclimated to the laboratory conditions in seven 700 L tanks (49 fish per tank) with permanently aerated water. The fish were kept under a 14:10 light–dark cycle, at a water temperature of 18 °C. The water quality parameters were as follows: dissolved oxygen concentration 9.0 mg/L, pH 7.6–7.7, water hardness 186 mg CaCO_3_/L. Heavy metal concentrations in water were as follows: 0.003 of mg Cu/L, 0.01 of mg Zn/L, 0.025 of mg Fe/L, and 0.0045 of mg Cd/L. The fish were fed commercial dry pellets daily (3% of their body weight). During the 3-month acclimation period and the experiment, the fish were maintained on the same diet regimen. The feed was comprised of 37% crude protein, 12% crude fat, and 32.5% carbohydrate. After the acclimation, the fish were randomly assigned to seven groups: control group—Cd-free water, Mel group—the fish were implanted with melatonin, blank group—the fish were sham-injected, 0.4 mg Cd/L + Mel group—the fish were implanted with melatonin and exposed to cadmium in water, 0.4 mg Cd/L group—the fish were exposed to cadmium in water, 4.0 mg Cd/L + Mel group—the fish were implanted with melatonin and exposed to cadmium in water, and 4.0 mg Cd/L group—the fish were exposed to cadmium in water. The cadmium concentrations used in the study were chosen considering the concentrations of Cd found in surface waters (between 1 and over 16 mg/L) [21,22]. The implants contained 18 g melatonin (Ceva Santè Animale, Libourne, France) and were placed intramuscularly under the dorsal fin. A number of other studies have shown that the blood concentration of melatonin in fish following the implantation of melatonin remains high (at around 1 ng/mL) for 5 months [23,24]. The procedure used to implant melatonin in the fish was carried out based on studies by Porter et al. [25] and Mazurais et al. [26]. The stock solutions of Cd were prepared by dissolving the necessary amount of cadmium chloride (CdCl_2_·2.5H_2_O, Avantor Performance Materials Poland S.A., Gliwice, Poland) in distilled water. The water in the tanks was renewed every 2 days. The analysis of cadmium concentrations in the water samples collected during the experiment showed the following mean levels of the metal: control, Mel and blank groups—0.006 mg/L (± 0.001), group 0.4 mg Cd/L + Mel—0.34 mg/L (± 0.05), group 0.4 mg Cd/L—0.41 mg/L (± 0.04), group 4.0 mg Cd/L + Mel—3.97 mg/L (± 0.49), and group 4.0 mg Cd/L—4.02 mg/L (± 0.39).

The control, Mel, and blank (not exposed to Cd) groups were kept under the same conditions during the 13 weeks of the experiment. After 7 weeks of exposure, each Cd-exposed group was divided into two groups of fish. One of them remained under the same conditions, while the other (groups 0.4 mg Cd/L + Mel-dep, 0.4 mg Cd/L-dep, 4.0 mg Cd/L + Mel-dep, and 4.0 mg Cd/L-dep) was transferred to Cd-free water for a depuration period, which lasted until the end of the experiment (next 6 weeks) (Table 1). The experiments were performed in accordance with the research protocols approved by the Local Animal Ethics Committee in Cracow, Poland (agreement no 97/V/2013).

### 2.2. Mortality, Body Weight and Behaviour

The mortality of the fish was monitored by recording all fish deaths during the entire experiment. Each fish from each tank was weighed before exposure and after weeks 1, 4, 7, 10, and 13 of exposure. Then, the mean body weight (scale type WPS 110/C/2, RADWAG, Radom, Poland) for the entire group was calculated. Every day during the feeding and every two days before water renew behavioral observations were conducted. Changes in the activity of the fish were assessed for anomalies, such as deficient movement coordination and with the decreased swimming behavior. Fish response to feeding as well as the feed consumption was observed to assess changes in fish appetite.

### 2.3. Heavy Metal Determination

Muscle tissue was dissected from 10 randomly caught fish from each group before exposure and after weeks 1, 4, 7, 10, and 13 of the experiment. Samples of muscles (about 5 g) were collected in order to determine Cd, Zn, Cu, and Fe levels. The preparation of fish tissues for heavy metal analysis and metal determination was presented elsewhere [27]. Metal levels in muscle tissue were analyzed by atomic absorption spectrometry using a Model ATI UNICAM 929 (Unicam Ltd., Cambridge, England) [28]. The results were expressed as milligrams of Cd, Zn, Cu, and Fe per kilogram of wet tissue weight (ww).

### 2.4. Bioconcentration Factor (BCF)

The bioconcentration factor (BCF) of heavy metals in Prussian carp was calculated as the ratio between the cadmium concentration in fish muscle tissue (C_tissue_) and its concentration in water (C_water_) [10].

BCF = C_tissue_ (mg/kg wet tissue)/C_water_ (mg/L) (1)

### 2.5. Statistical Analysis

The results of the analysis were expressed as a mean ± standard error of the mean (SEM). The results were analyzed using repeated-measure ANOVA (for body weight measures) and one-way ANOVA (for heavy metals concentration results), and then a Student’s *t*-test was used to determine significant differences between the means for the control and experimental groups, between groups in consecutive months of exposure, and within the same group in subsequent sampling weeks. The relationship between cadmium, zinc, copper, and iron levels in muscle tissue and the Cd concentration used for the exposure was calculated using Pearson’s correlation coefficients. The differences between the means were determined as significant for *p* < 0.05.

## 3. Results

### 3.1. Mortality, Body Weight and Behaviour

The highest mortality (3%) was found in the group exposed to the higher cadmium concentration (1 fish in tenth week and 2 fish in thirteenth week). No deaths were recorded in other groups. Melatonin-treated fish remained healthy during the entire observation period. At the beginning of the experiment, there were no differences in the mean body weight between the groups In the control groups and in the 0.4 mg Cd/L + Mel, 0.4 mg Cd/L and 4.0 mg Cd/L + Mel groups, and no significant change in the mean body weight was observed during the entire observation period, whereas a significant decrease in body weight was observed in the 4.0 mg Cd/L group after 10 and 13 weeks of exposure (146.6 and 165.5 g, respectively) (Table 2). After 3 weeks of depuration, the body weight of fish in the 4.0 mg Cd/L group was statistically significantly lower (137.0 ± 13.40 g) compared with other groups. After 6 weeks of depuration, a significant increase in the body weight of fish in that group was observed (196.2 ± 16.2 g) and it was found that the body weight of these fish was statistically significantly higher compared with that of the fish that were exposed to Cd throughout the duration experiment (164.6 ± 19.6 g). Melatonin was not found to have an impact on the increase in body weight during the depuration period (Table 2).

Behavioral observations suggest that fish in the control, blank, and Mel groups swam normally, with no abnormalities observed, throughout the entire experiment. In turn, after one week of exposure, fish exposed to the higher concentration of cadmium (4.0 mg Cd/L) showed behavioral changes such as erratic swimming and sinking. The presence of uneaten feed on the tank bottom in this group before each water renewal suggested lack of appetite in fish. The intensity of this behavior increased with the duration of exposure. In contrast, fish in the 4.0 mg Cd/L + Mel group swam normally and had a normal appetite.

### 3.2. Cd Accumulation in Muscle

The results of the analysis of cadmium levels in the muscle tissue of Prussian carp females after weeks 0, 1, 4, 7, 10, and 13 of exposure to different cadmium concentrations are presented in Table 3. A statistically significant (*p* < 0.05) increase in Cd levels in muscle tissue compared with sample 0 (baseline) was observed as early as in the first week of exposure. The increase continued until the end of exposure. The statistically significant positive correlation coefficient obtained confirmed the correlation between cadmium accumulation and the exposure concentration of this metal (Table 4). The highest level of cadmium (0.61 mg/kg) was found after week 10 of exposure (Table 3). In the group of fish exposed to the higher concentration of Cd (4.0 mg Cd/L), Cd levels were significantly higher (*p* < 0.05) compared with the control and other groups at all sampling times. In the group of fish that received melatonin implants (4.0 mg Cd/L + Mel), Cd levels in muscle were significantly lower (*p* < 0.05) compared with the group exposed to the higher concentration of cadmium (4.0 mg Cd/L) during the entire observation period, except for week 7. Pearson’s correlation coefficients were calculated to determine the correlation between the concentration of Cd in water and Cd levels in muscle tissue during the period of exposure. Positive correlations were recorded from week 1 until the end of exposure (Table 4).

The results of the analysis of Cd levels after 7 weeks of exposure and 6 weeks of depuration are presented in Table 3. After 3 weeks of depuration, a statistically significant (*p* < 0.05) increase in Cd levels was observed in the muscle of fish exposed to the higher concentration of the metal (4.0 mg Cd/L-dep), whereas a statistically significant decrease in muscle Cd levels was observed in the 4.0 mg Cd/L + Mel-dep group (Table 3). After 6 weeks of depuration of fish exposed to the higher concentration of Cd, a decrease in muscle cadmium levels was observed compared with the levels observed after 3 weeks of depuration. The positive Pearson’s correlation coefficient found after 3 and 6 weeks of depuration confirmed that Cd levels in muscle were statistically significantly correlated with Cd concentration in water (Table 4).

### 3.3. Zn Accumulation in Muscle

The results of the analysis of zinc levels in the muscle of Prussian carp are presented in Table 3. In the group of fish exposed to the higher concentration of cadmium, a statistically significant increase (*p* < 0.05) in Zn levels in muscle tissue compared with other groups was observed after 7 and 10 weeks of exposure. In the group of melatonin-treated fish (4.0 mg Cd/L + Mel), Zn levels in muscle tissue between weeks 4 and 10 of exposure were statistically significantly lower (*p* < 0.05) compared with the 4.0 mg Cd/L group. The positive correlation coefficients found after weeks 1, 4, 7, 10, and 13 of exposure confirm a positive correlation between Zn levels and Cd levels in muscle tissue (Table 4).

After 6 weeks of depuration, a statistically significant difference in Zn levels was observed between the 4.0 mg Cd/L group and the 4.0 mg Cd/L + Mel group (Table 3). During the depuration period, Zn levels in the muscle of the fish studied were also positively correlated with muscle Cd levels (Table 4).

### 3.4. Cu Accumulation in Muscle

The results of the analysis of Cu levels in the muscle of Prussian carp females are presented in Table 5. After 1 week of exposure, Cu levels in the muscle of fish exposed to the higher Cd concentration in water (4.0 mg Cd/L and 4.0 mg Cd/L + Mel) were statistically significantly higher compared with the control and other groups. In turn, a statistically significant decrease (*p* < 0.05) in Cu levels in the muscle of fish exposed to the higher concentration of Cd (4.0 mg Cd/L) compared with the control was observed after weeks 10 and 13 of exposure (Table 5). The decrease in Cu levels was significantly correlated with Cd levels in the muscle of Cd-exposed fish. This was confirmed by statistically significant negative correlation coefficients between Cu levels and Cd levels in muscle tissue after 10 and 13 weeks of exposure (Table 4). After 13 weeks of exposure, a statistically significant decrease (*p* < 0.05) was observed in muscle Cu levels in the 4.0 mg Cd/L group. Moreover, a statistically significant difference in Cu levels was observed between the groups of Cd-exposed fish that did not receive melatonin implants and those that were exposed to cadmium and treated with melatonin (Table 5).

During the period of depuration, an increase in Cu levels, accompanying a decrease in Cd levels in muscle tissue, was observed (Table 3 and Table 5). The negative correlation between Cu levels and Cd levels in the muscle of fish subjected to depuration was confirmed by the negative correlation coefficient found after 3 weeks of depuration (Table 4).

### 3.5. Fe Accumulation in Muscle

The results of the analysis of iron levels in the muscle of Prussian carp are presented in Table 5. In the control, blank, and Mel groups, Fe levels in muscle tissue ranged between 4.41 ± 0.29 and 10.74 ± 0.5 mg/kg. A similar balance in iron levels was also observed in the groups of fish exposed to 0.4 mg Cd/L. In the 4.0 mg Cd/L group, Fe levels after 4 and 10 weeks of exposure were significantly higher compared with the melatonin-treated group (Table 5). In turn, significantly higher Fe levels were observed in the 4.0 mg Cd/L-Mel group after weeks 1 and 7 of exposure. Positive correlations between the levels of Fe and Cd in muscle tissue were observed after weeks 4 and 10 of exposure (Table 4).

During the depuration period, a gradual decrease in muscle Fe levels was observed in almost all Cd-exposed groups (0.4 mg Cd/L, 0.4 mg Cd/L + Mel, 4.0 mg Cd/L + Mel). The 4.0 mg Cd/L group was the only group that, after 3 weeks of depuration, displayed an increase in Fe levels (Table 5). After the last week of depuration, an increase in Fe levels was observed in the Cd-exposed group compared with other groups, which was confirmed by a positive correlation between Fe levels and Cd levels in muscle tissue (Table 4).

### 3.6. BCF

Bioconcentration factor (BCF) values for Cd in muscle tissue are presented in Table 6. In the present study, a statistically significant (*p* < 0.05) increase in BCF values was found in all Cd-exposed groups, with the values increasing along with the duration of exposure. Highest BCF values were observed in fish exposed to the lower concentration of cadmium—0.4 mg/L. It was also found that in melatonin-treated fish, BCF values were lower compared to Cd-exposed fish that did not receive melatonin implants (Table 6).

## 4. Discussion

Currently, there is an increasing interest among researchers in studying the uptake of heavy metals by aquatic organisms, which reflects the increasing threat of heavy metal contamination of the aquatic environment caused by human activity [16]. As shown by Djedjibegovic et al. [6], fish and seafood, which are valuable protein sources in a healthy diet, can bioaccumulate heavy metals. Those metals are transferred to higher levels in the food chain through biomagnification and can have a harmful effect on the health of aquatic organisms and people.

Cadmium is a priority substance, particularly harmful to living organisms. This is confirmed by the results of the present study, which found a decrease in body weight and a higher mortality rate in the group of fish exposed to the higher concentration of cadmium (Table 2). The inhibitory effect of cadmium on fish growth may result from the impaired intake and absorption of food. This was confirmed by the presence of uneaten food on the tank bottom on the next day after the feeding, what may suggest the loss of appetite in fish exposed to increased cadmium concentrations. The negative impact of cadmium on the growth, body weight, and mortality of fish has also been reported by other authors [29,30]. While the present study found no clear impact of melatonin on the body weight of the fish studied, melatonin certainly improved the survival of the fish as no deaths were recorded in the Cd-exposed fish that received continuous-release melatonin implants. It may be assumed that the immunostimulating effect of melatonin helped reduce the mortality of the fish [31].

One of the earliest reactions of fish to environment pollution (disturbance) are changes in their behavior. Reactions such as surfacing, slow movement, increased opercular movements, and increased mucus secretion have been reported in various fish species exposed to cadmium [30,32]. Similar behavioral changes were also observed in the present study in the group of fish exposed to the higher concentration of cadmium, with the most intense abnormalities observed in weeks 10 and 13 of exposure. The other groups did not present any changes in behavior or locomotor activity.

Fish accumulate Cd in their tissues mainly from water and food. Observations show that cadmium enters the body mainly through the respiratory system and, to a lesser extent, through the digestive tract [33]. In the present study on Prussian carp, a significant increase in cadmium levels in muscle tissue was observed after just one week of exposure in the groups of fish exposed to the higher concentration of this metal in water. It was also found that Cd accumulation continued to increase gradually with the duration of exposure. The highest muscle cadmium levels were observed after week 10 of exposure (0.61 mg/kg) (Table 3). Furthermore, during the same period, the highest BCF value was recorded in the group exposed to the higher concentration of cadmium (Table 6). Similar observations were made by Malekpouri et al. [34], who found that Cd accumulation in the muscle of carp (*Cyprinus carpio*) grew along with the increasing concentration of the metal in water and duration of cadmium exposure. Al-Sawafi et al. [16] found a similar increase in Cd accumulation in the muscle of zebrafish (*Danio rerio*) exposed to this metal. In the present study, cadmium levels remained at 0.02–0.61 mg/kg during the entire period of exposure and significantly exceeded the maximum permissible concentration of cadmium in the muscle of edible fish for the European Union, which is 0.05 mg/kg [35]. The Cd levels observed in this study also exceeded the recommendations of the Food and Agriculture Organization/ World Health Organization (FAO/WHO), which indicate that the tolerable cadmium intake for adults is around 0.4–0.5 mg/week and the acceptable daily intake is 60–70 μg. Cadmium has a wide range of effects on the function of fish muscle tissue. It has been reported to impair the function of muscle fibers (fiber excitation-–contraction coupling), upregulate various proto-oncogenes, reduce protein levels, modify enzymatic activity, deplete glycogen reserves, and reduce lipid levels [36,37]. High cadmium levels may cause changes in the muscle structure. Avallone et al. [36] found that cadmium disrupts the sarcomeric pattern and causes structural disorganization, especially disassembly of muscle myofibrils. Exposure to this heavy metal has also been reported to cause the impairment of muscle function related to mitochondrial damage, which reduces swimming performance [38]. In the present study, similar observations, i.e., weakness and erratic swimming, were made after just one week of the experiment in fish exposed to the higher concentration of cadmium. Those abnormalities intensified with the duration of exposure.

The administration of melatonin to Prussian carp females exposed to cadmium significantly reduced Cd accumulation in the muscle of the fish. In the case of fish exposed to the higher concentration of Cd, a decrease in Cd accumulation was observed as early as in the first week of exposure and this effect continued until week 13 (Table 3). This was also confirmed by lower BCF values in the Cd-exposed fish that were treated with melatonin compared with the Cd-exposed fish that did not receive melatonin implants (Table 6). According to our knowledge, this study is the first report on the protective effects of melatonin against the accumulation of cadmium in the muscle tissue of fish (Prussian carp) exposed to this metal. Melatonin is an antioxidant and a free-radical scavenger capable of removing reactive oxygen species (ROS). This is particularly important since the hormone can cross all morpho-physiological barriers in the body due to its distinct physical and chemical properties that allow it to penetrate cell barriers and nuclei [39,40]. Moreover, due to its anti-inflammatory and anti-apoptotic effects, melatonin supports muscle healing after injury [18]. Mitochondria, which are numerous in muscles, are the target of melatonin, which maintains them efficiently, scavenging free radicals and reducing oxidative damage [20,41]. The findings from the present study suggest that the co-administration of melatonin may effectively prevent the accumulation of cadmium and thus protect against the toxic effect of the metal on the muscle of Prussian carp.

Being similar to such biometals as zinc, copper, and iron, cadmium that accumulates in a healthy organism disrupts their homeostasis and biological functions. To date, no studies have been carried out into changes in the levels of essential trace elements such as Zn, Cu, and Fe during long-term exposure to Cd and/or melatonin in Prussian carp. Studies on the toxic effect of cadmium on organisms have shown that it interacts with metals such as iron, copper, and zinc [33,42,43]. This interaction is due to the similar physical and chemical properties of those metals. Cadmium has been reported to significantly affect the levels of Zn, Fe, and Cu in blood, depending on its concentration and/or duration of exposure [43]. Furthermore, the homeostasis of Cu, Zn, and Fe plays a key role in the toxic effect of cadmium, leading to a general threat to basic cellular functions [42].

The present study found an increase in Zn levels in the muscle of Prussian carp, accompanying an increase in Cd levels, in the group of fish exposed to 4.0 mg Cd/L compared to other groups in week 4 of exposure. The increase continued until week 10 (Table 3). This confirms the positive correlation between Zn levels and Cd absorption in muscle tissue (Table 4), which has also been observed by Astani et al. [44] and Petrea et al. [45]. Changes in the levels of zinc in tissues and organs following exposure to cadmium have also been reported by Lane et al. [42] and Bulat et al. [14]. These changes result from the relation between Cd^2 +^ and Zn^2 +^ ions for transporter-mediated entry into the cell and binding to intracellular sites [46,47].

The present study found that copper levels in the fish studied decreased, in parallel with an increase in Cd levels, along with the duration of exposure to cadmium. This confirms the negative correlation coefficients between Cd levels and Cu levels in muscle tissue recorded after weeks 10 and 13 of exposure (Table 5). Similarly, studies by Lane et al. [42] and Orisakwe et al. [48] found a negative correlation between Cd and Cu levels. Cadmium is a strong inhibitor of Cu metabolism that exhibits strong antagonistic activity. Moreover, a decrease in copper levels in muscle tissue disrupts the functioning of mitochondria and production of energy in muscles, which reduces their capacity to perform work [49,50]. The present study also found such abnormalities as slowness of movement and difficulty swimming, which intensified as the period of exposure increased, in the group of fish exposed to the higher concentration of cadmium. Those locomotor problems may have also been due to skeletal muscle hypoxia. Cu is known to play an important role in red blood cell production. The displacement of Cu by cadmium that accumulates in the body may disrupt hematopoietic processes, which may lead to anemia, and skeletal muscle and myocardial hypoxia [51,52].

In addition to copper and zinc, another bioelement that was analyzed in the present study was iron. Iron is an important component of various metalloproteins, such as hemoglobin and myoglobin, and plays a key role in the optimal functioning of skeletal muscle tissue [53,54]. The findings from our study clearly show that exposure to cadmium affects the levels of Fe in the muscle of Cd-exposed Prussian carp (Table 5). Iron levels show an inconsistent (alternating) pattern (decrease, increase, decrease, increase). In the present study, Fe levels in the muscle of Prussian carp exposed to Cd at a concentration of 4.0 mg/L increased in weeks 4 and 10 of exposure and decreased in weeks 1 and 7 (Table 5). The results of this study corroborate the conclusions of several other studies, which found that the distribution of Fe in tissues depends on the duration of exposure to cadmium and its concentration as well as that cadmium may cause the redistribution of Fe and its exchange in iron-dependent enzymes and proteins [55,56]. Both iron deficiency and iron excess (overload) have harmful effects on cellular mechanisms involved in the production of energy [54].

Transition metal ions are key elements of various biological processes, from oxygen transport to hypoxia detection. Therefore, their homeostasis is maintained within tight limits through strictly regulated absorption, storage, and excretion mechanisms. Disruption of the homeostasis of such metal ions as Zn, Cu, and Fe can lead to the uncontrolled production of ROS, which may impair the effectiveness of skeletal muscle tissue function [54,57,58]. One of the body’s natural free-radical scavengers and substances that affect the metabolism (homeostasis) of elements in tissues is the melatonin hormone [59,60]. To date, little has been known about the Zn, Cu, and Fe status under the conditions of simultaneous exposure to both Cd and melatonin. In our study, melatonin significantly regulated (affected) the levels of microelements (Table 3 and Table 5). The presence of melatonin stabilized the muscle levels of the microelements studied.

The abovementioned findings suggest that melatonin plays a role in maintaining the homeostasis of elements in fish exposed to the toxic effects of cadmium. This is confirmed by studies indicating that melatonin and its precursors, tryptophan and serotonin, interact with certain metals that are of biological importance (Zn, Cu, Fe) [59,61,62]. Melatonin creates complexes with cadmium, copper, iron, and zinc, which suggests that it is involved in the maintenance of homeostasis and detoxification of metals in the biological system [61,63].

Melatonin supplementation may play an important role in preventing the possible toxic effects of cadmium and regulating the homeostasis of trace elements in the case of exposure to cadmium.

It is not only the accumulation but also the elimination of metals from fish tissue that is determined by several factors. These include the duration of depuration, age of the fish, temperature, interactions with other heavy metals, metabolic activity of the fish and the biological half-life of the metal concerned. Fish eliminate metals through the gills, urine, bile, and mucus [64]. In our study, the elimination efficiency of cadmium accumulated over 7 weeks of exposure was mainly determined by the duration of the period of depuration. Three weeks after cessation of exposure to the higher concentration of cadmium (4.0 mg/L), a further increase in the levels of this metal in fish muscle was observed. This was confirmed by the (significantly) positive Pearson’s correlation coefficient recorded at that time point. It was only 6 weeks after the end of exposure that effective Cd elimination from muscle tissue was observed (Table 3). Likewise, several earlier studies found that the elimination of cadmium from the muscle of the fish studied was slow and preceded by ongoing accumulation after the end of exposure [64,65].

Melatonin treatment during the period of depuration resulted in a decrease in muscle Cd levels as early as 3 weeks after the end of exposure. The decrease was significantly lower compared with the groups of fish that did not receive melatonin implants (Table 3).

During the depuration period, changes in the muscle levels of heavy metals such as Zn, Cu, and Fe were also observed. An increase in Cu and Fe levels was recorded after the third and sixth weeks of cadmium elimination from muscle tissue. This was probably due to the re- accumulation of cadmium released from other tissues during that period. In turn, zinc levels in the muscle of the fish studied did not change during the period of elimination, which suggests that the concentration of this metal is regulated through a different mechanism (Table 3 and Table 5). A similar finding was made in a study on Redbelly tilapia (*Tilapia zillii*) [66].

## 5. Conclusions

The exposure of Prussian carp females to cadmium in water resulted in dose- and exposure duration-dependent changes, as manifested in the mortality of the fish, changes in their locomotor activity and body weight, accumulation of Cd in muscle tissue, and disruption of the homeostasis of Fe, Cu, and Zn in the muscle of the fish. The administration of melatonin (a safe nutraceutical) to fish exposed to Cd statistically significantly prevented (inhibited) the accumulation of cadmium in muscle tissue, which suggests that melatonin can prevent damage to skeletal muscle tissue caused by the toxic effects of cadmium. The administration of melatonin to Cd-exposed fish also mitigated the disruption of their microelement homeostasis as compared with Cd-exposed fish that were not treated with melatonin. Interactions between cadmium and/or melatonin and bioelements may take place at different stages of the absorption, distribution, and excretion of bioelements and Cd, and at the level of biological functions of essential elements. Thus, an analysis of molecular mechanisms is required. Further studies are needed to better understand the effect of melatonin on Cd turnover as well as the interaction of Cd and melatonin with the metabolism of Zn, Cu, and Fe.

## Figures and Tables

**Table 1:** The configuration of treatment groups and cadmium doses in water during the 13-week exposure period and the following 6-week exposure or depuration period.

Group	Control	Mel	Blank	0.4 mg Cd/L + Mel		0.4 mg Cd/L		4.0 mg Cd/L + Mel		0.4 mg Cd/L	
Cd dose in water (mg/L) during 1–7-week period	-	-	-	0.4		0.4		4.0		4.0	
Number of fish at the beginning [*n*] (per tank)	49	49	49	49		49		49		49	
Group during 7–13 weeks of experiment	control	Mel	blank	0.4 mg Cd/L + Mel	0.4 mg Cd/L + Mel-dep	0.4 mg Cd/L	0.4 mg Cd/L -dep	4.0 mg Cd/L + Mel	4.0 mg Cd/L + Mel-dep	4.0 mg Cd/L	4.0 mg Cd/L -dep
Cd dose in water (mg/L) during the 7–13-week period	-	-	-	0.4	-	0.4	-	4.0	-	4.0	-
Number of fish at the beginning of the 7th week of the exposure/depuration	14/14	14/14	14/14	14	14	14	14	14	14	14	14

“Mel”: group of fish with melatonin implant; “-“: Cd was not added into the water.

**Table 2:** Body weight (g) of female Prussian carp during 13 weeks of fish exposure to different doses of cadmium and 6 weeks depuration.

Week	Control	Mel	Blank	0.4 mg Cd/L + Mel	0.4 mg Cd/L	4.0 mg Cd/L + Mel	4.0 mg Cd/L	0.4 mg Cd/L + Mel-Dep	0.4 mg Cd/L−Dep	4.0 mg Cd/L + Mel-Dep	4.0 mg Cd/L−Dep
Exposure								Depuration			
0	217.0 ± 14.76 Aa	205.1 ± 12.73 Aa	198.0 ± 8.26 Aab	226.6 ± 16.03 Aa	196.6 ± 7.79 Aa	210.9 ± 13.88 Aa	217.0 ± 14.63 Aa	NT	NT	NT	NT
1	218.1 ± 14.64 ABa	206.9 ± 10.25 Aab	210.3 ± 13.10 Aab	210.3 ± 11.17 Aa	212.3 ± 18.14 Aa	174.6 ± 7.05 Ba	213.0 ± 1.27 Aa	NT	NT	NT	NT
4	227.6 ± 17.87 Aa	218.6 ± 13.51 Aab	201.1 ± 9.90 ABa	216.7 ± 15.12 Aa	165.7 ± 10.47 Ba	185.0 ± 11.74 Aa	183.1 ± 15.22 ABab	NT	NT	NT	NT
7	205.4 ± 10.00 ABa	210.6 ± 15.35 ABab	209.4 ± 10.21 ABab	210.1 ± 6.73 Aa	184.0 ± 13.66 ABa	172.6 ± 14.64 Ba	168.3 ± 13.65 ABb	NT	NT	NT	NT
10	183.6 ± 13.69 ABa	199.01 ± 7.44 ABab	167.3 ± 20.19 ABa	205.7 ± 13.67 Aa	168.6 ± 11.29 ABa	165.4 ± 5.75 Ba	146.6 ± 14.51 ABb	199.1 ± 11.24 Aa	192.9 ± 11.86 Aa	165.4 ± 8.69 CBa	137.0 ± 13.40 Ba
13	217.4 ± 16.12 ABCa	248.0 ± 19.01 ABb	235.4 ± 14.41 ABb	242.3 ± 12.32 Aa	200.0 ± 10.44 CBa	182.6 ± 10.59 Ca	165.5 ± 19.69 Cab	209.1 ± 18.73 ABa	220.0 ± 11.72 ABa	179.3 ± 12.82 Ba	196.2 ± 16.20 ABb

NT: not tested, capital letters denote statistically significant differences (*p* < 0.05) between the groups in the same time exposure (row), while small letters indicate significant differences in the groups between successive weeks of the exposure (column).

**Table 3:** Comparison of Cd and Zn levels (mg/kg ww) in the muscles of female Prussian carp during 13 weeks of fish exposure to different doses of cadmium and 6 weeks depuration.

Week	Control	Mel	Blank	0.4 mg Cd/L + Mel	0.4 mg Cd/L	4.0 mg Cd/L + Mel	4.0 mg Cd/L	0.4 mg Cd/L + Mel-Dep	0.4 mg Cd/L−Dep	4.0 mg Cd/L + Mel-Dep	4.0 mg Cd/L−Dep
Cd in muscle (mg/kg wet weight)											
Exposure								Depuration			
0	0.01 ± 0.001 Aa	0,02 ± 0.001 Aa	0.01 ± 0.001 Aa	0.02 ± 0.001 Aa	0.01 ± 0.001 Aa	0.02 ± 0.005 Aa	0.02 ± 0.001 Aa	NT	NT	NT	NT
1	0.01 ± 0.002 Aa	0.02 ± 0.003 Aa	0.02 ± 0.002 Aab	0.02 ± 0.003 Aa	0.02 ± 0.001 Aa	0.04 ± 0.003 Bb	0.06 ± 0.006 Cb	NT	NT	NT	NT
4	0.02 ± 0.001 Abc	0.03 ± 0.003 Ab	0.02 ± 0.003 Bab	0.04 ± 0.004 BCb	0.04 ± 0.004 Cb	0.09 ± 0.006 Dc	0.10 ± 0.008 Ec	NT	NT	NT	NT
7	0.02 ± 0.001 Ac	0.03 ± 0.004 ABb	0.03 ± 0.002 Abc	0.04 ± 0.003 BCb	0.04 ± 0.002 Cb	0.21 ± 0.009 Dd	0.23 ± 0.01 Dd	NT	NT	NT	NT
10	0.03 ± 0.001 Ad	0.03 ± 0.002 Ab	0.02 ± 0.002 Bab	0.05 ± 0.005 Cb	0.07 ± 0.005 Dc	0.36 ± 0.04 Ee	0.61 ± 0.06 Fe	0.02 ± 0.002 BCab	0.02 ± 0.002 Cab	0.25 ± 0.01 Dd	0.32 ± 0.03 Ef
13	0.04 ± 0.002 ABc	0.05 ± 0.005 Ac	0.03 ± 0.002 BCc	0.03 ± 0.003 Cab	0.08 ± 0.005 Dc	0.38 ± 0.02 Ee	0.56 ± 0.05 Fe	0.02 ± 0.003 Bab	0.04 ± 0.004 Aab	0.12 ± 0.008 Dc	0.14 ± 0.01 Dc
**Zn in muscle (mg/kg wet weight)**											
**Exposure**								**Depuration**			
0	11.89 ± 0.58 Aa	11.89 ± 0.61 Aa	11.06 ± 0.65 Aab	11.89 ± 0.61 Aa	10.94 ± 0.50 Aa	10.94 ± 0.69 Aac	11.06 ± 0.47 Aa	NT	NT	NT	NT
1	10.82 ± 0.33 ABa	10.07 ± 0.38 Aa	11.95 ± 0.67 ABCab	11.21 ± 0.45 ABa	12.11 ± 0.68 Ba	13.34 ± 0.77 Cb	12.04 ± 0.65 ABCab	NT	NT	NT	NT
4	11.32 ± 0.46 Aa	11.34 ± 0.67 ABa	11.62 ± 0.71 ABab	10.91 ± 0.72 ABa	11.92 ± 0.61 ABa	10.88 ± 0.49 Aa	13.36 ± 0.61 Bb	NT	NT	NT	NT
7	9.95 ± 0.50 Aa	10.97 ± 0.73 ABa	10.39 ± 0.51 Aa	10.92 ± 0.31 Aa	10.16 ± 0.43 Aa	13.56 ± 0.72 Bb	16.95 ± 0.68 Cc	NT	NT	NT	NT
10	11.54 ± 0.56 Aa	11.00 ± 1.03 Aa	13.63 ± 0.98 Ab	10.94 ± 0.65 Aa	13.25 ± 0.72 Ab	13.04 ± 0.55 Ab	18.12 ± 1.00 Bc	11.77 ± 0.72 Aa	12.04 ± 0.51 Aa	14.22 ± 0.68 Ab	13.49 ± 0.82 Ab
13	10.13 ± 0.48 Aa	10.59 ± 0.77 Aa	10.11 ± 0.50 Aa	10.53 ± 0.51 ABa	10.68 ± 0.55 ABa	11.65 ± 0.77 ABab	12.91 ± 0.79 Bab	8.49 ± 0.72 Ab	10.11 ± 1.14 Aa	12.01 ± 0.60 Bab	14.27 ± 1.10 Cb

NT: not tested, NS: not significant, capital letters denote statistically significant differences (*p* < 0.05) between the groups in the same time exposure (row), while small letters indicate significant differences in the groups between successive weeks of the exposure (column).

**Table 4:** Pearson’s correlation coefficients (r) for the relationship of cadmium concentration in muscles of fish and Cd dose in water as well as between Cd concentration in muscles and other heavy metals (Zn, Cu, Fe) level in muscles of the same fish, during the exposure and depuration periods, in consecutive weeks of the experiment.

Week	Heavy Metals	Exposure		Deputation	
		Cd in Water	Cd in Muscles	Cd in Water	Cd in Muscles
1	Cd	0.71 ***	-	-	-
	Zn	-	0.34 *	-	-
	Cu	-	0.46 ***	-	-
	Fe	-	NS	-	-
4	Cd	0.88 ***	-	-	-
	Zn	-	0.29 *	-	-
	Cu	-	0.45 **	-	-
	Fe	-	0.43 **	-	-
7	Cd	0.89 ***	-	-	-
	Zn	-	0.76 ***	-	-
	Cu	-	NS	-	-
	Fe	-	NS	-	-
10	Cd	0.92 ***	-	0.48 ***	-
	Zn	-	0.60 ***	-	0.34 *
	Cu	-	−0.32 *	-	−0.32 *
	Fe	-	0.52 ***	-	NS
13	Cd	0.71 ***	-	0.58 ***	-
	Zn	-	0.50 ***	-	0.58 ***
	Cu	-	−0.47 ***	-	NS
	Fe	-	NS	-	0.56 ***

* *p* < 0.05; ** *p* < 0.01; *** *p* < 0.001; NS-no statistically significant correlation; - not tested.

**Table 5:** Comparison of Cu and Fe levels (mg/kg ww) in the muscles of female Prussian carp during 13 weeks of fish exposure to different doses of cadmium and 6 weeks depuration.

Week	Control	Mel	Blank	0.4 mg Cd/L + Mel	0.4 mg Cd/L	4.0 mg Cd/L + Mel	4.0 mg Cd/L	0.4 mg Cd/L + Mel-dep	0.4 mg Cd/L-Dep	4.0 mg Cd/L + Mel-Dep	4.0 mg Cd/L-Dep
Cu in muscle (mg/kg wet weight)											
Exposure								Depuration			
0	0.44 ± 0.04 Aac	0.44 ± 0.03 Aa	0.44 ± 0.03 Aac	0.47 ± 0.04 Aa	0.43 ± 0.03 Aa	0.43 ± 0.03 Aa	0.44 ± 0.04 Aa	NT	NT	NT	NT
1	0.31 ± 0.02 Ab	0.36 ± 0.02 Ab	0.36 ± 0.02 Bac	0.38 ± 0.02 Ba	0.34 ± 0.03 Bac	0.45 ± 0.04 Cab	0.42 ± 0.04 Cab	NT	NT	NT	NT
4	0.34 ± 0.02 ACa	0.24 ± 0.02 BCb	0.31 ± 0.04 Aab	0.20 ± 0.02 BCb	0.23 ± 0.02 Bb	0.28 ± 0.03 Cb	0.36 ± 0.03 ADa	NT	NT	NT	NT
7	0.24 ± 0.03 Ab	0.22 ± 0.01 Ab	0.25 ± 0.01 Ab	0.25 ± 0.03 Ab	0.15 ± 0.01 Bc	0.14 ± 0.01 Bc	0.24 ± 0.01 Ab	NT	NT	NT	NT
10	0.49 ± 0.03 Ac	0.55 ± 0.05 Aa	0.52 ± 0.03 Ac	0.43 ± 0.03 ABa	0.46 ± 0.03 ABa	0.44 ± 0.05 ABab	0.37 ± 0.03 Ba	0.45 ± 0.03 Ab	0.52 ± 0.03 Ab	0.50 ± 0.05 Ab	0.34 ± 0.04 Bb
13	0.41 ± 0.03 Aa	0.50 ± 0.03 BCa	0.38 ± 0.03 Aac	0.52 ± 0.03 Ba	0.33 ± 0.02 ADa	0.40 ± 0.04 Ca	0.28 ± 0.01 Db	0.35 ± 0.03 Cc	0.43 ± 0.03 ABCc	0.41 ± 0.01 ABCb	0.46 ± 0.03 ABCc
**Fe in muscle (mg/kg wet weight)**											
**Exposure**								**Depuration**			
0	9.46 ± 0.68 Aac	9.45 ± 0.67 Aac	9.46 ± 0.066 Aa	9.45 ± 0.68 Aa	9.31 ± 0.63 Aac	9.03 ± 0.88 Aac	9.46 ± 1.07 Aac	NT	NT	NT	NT
1	8.24 ± 0.64 Aa	8.65 ± 0.41 Aab	10.78 ± 0.50 BCa	10.61 ± 0.69 BCa	9.99 ± 0.90 ABa	11.79 ± 0.86 Cab	9.58 ± 0.51 ABa	NT	NT	NT	NT
4	10.74 ± 0.47 Ac	10.7 ± 1.08 Aa	10.66 ± 0.67 Aa	7.39 ± 0.40 Bb	9.77 ± 0.39 Aa	10.42 ± 0.52 Aabc	14.33 ± 0.63 Bb	NT	NT	NT	NT
7	4.41 ± 0.29 Ab	7.55 ± 0.40 Bb	9.80 ± 0.63 Ca	10.7 ± 0.60 Ca	9.15 ± 0.70 Cac	12.58 ± 0.81 Db	6.97 ± 0.81 ABcd	NT	NT	NT	NT
10	7.25 ± 0.74 ACa	8.47 ± 0.59 ABab	8.57 ± 0.83 ABb	6.29 ± 0.40 Cb	7.48 ± 0.42 ACc	8.57 ± 0.73 ACc	10.41 ± 0.77 Ba	6.10 ± 0.28 Bb	8.62 ± 0.69 Aab	8.51 ± 0.53 Ab	8.19 ± 0.77 Aa
13	5.04 ± 0.36 Ab	6.79 ± 0.74 ABb	5.02 ± 0.26 Ab	6.56 ± 0.53 ABb	5.17 ± 0.31 ABb	6.94 ± 0.47 Bd	5.68 ± 0.39 ABd	3.96 ± 0.46 Cc	5.71 ± 0.51 Ab	6.21 ± 0.34 Bc	8.06 ± 0.58 Da

NT: not tested, NS: not significant, capital letters denote statistically significant differences (*p* < 0.05) between the groups in the same time exposure (row), while small letters indicate significant differences in the groups between successive weeks of the exposure (column).

**Table 6:** Bioconcentration factors (BCF) in the muscles of female Prussian carp expos to different doses of cadmium for 1, 4, 7, 10, and 13 weeks.

Week	BCF			
	0.4 mg Cd/L + Mel	0.4 mg Cd/L	4.0 mg Cd/L + Mel	4.0 mg Cd/L
1	0.05 ± 0.009 Aa	0.04 ± 0.009 Aa	0.01 ± 0.009 Ba	0.02 ± 0.008 Ca
4	0.09 ± 0.010 Ab	0.11 ± 0.010 Ab	0.02 ± 0.008 Bb	0.03 ± 0.009 Bb
7	0.09 ± 0.010 Ab	0.10 ± 0.010 Ac	0.05 ± 0.009 Bc	0.06 ± 0.009 Bc
10	0.13 ± 0.011 Ab	0.18 ± 0.011 Bd	0.09 ± 0.009 Ad	0.15 ± 0.011 Cd
13	0,07 ± 0.009 Ac	0.19 ± 0.10 Bd	0.09 ± 0.100 Cd	0.14 ± 0.010 Dd

Capital letters denote statistically significant differences (*p* < 0.05) between the groups in the same time exposure (row), while small letters indicate significant differences in the groups between successive weeks of the exposure (column).

## Data Availability

All data are available from the authors’ database.
